# Optimizing Yarn Tension in Textile Production with Tension–Position Cascade Control Method Using Kalman Filter

**DOI:** 10.3390/s23125494

**Published:** 2023-06-11

**Authors:** Ahmed Neaz, Eun Ha Lee, Tae Hwan Jin, Kyung Chul Cho, Kanghyun Nam

**Affiliations:** 1 School of Mechanical Engineering, Yeungnam University, 280 Daehak-Ro, Gyeongsan 38541, Republic of Korea; ahmedneaz@yu.ac.kr; 2 Energy System Research Center, Korea Textile Machinery Convergence Research Institute, Gyeongsan 38542, Republic of Korea; eunha@kotmi.re.kr (E.H.L.); jinth@kotmi.re.kr (T.H.J.)

**Keywords:** roll-to-roll control, tension–position cascade control, control schematic with disturbance observer, signal processing with Kalman filter, robustness control

## Abstract

The production of textiles has undergone a considerable transformation, progressing from its primitive origins in hand-weaving to the implementation of contemporary automated systems. Weaving yarn into fabric is a crucial process in the textile industry that requires meticulous attention to output quality products, particularly in the tension control section. The efficiency of the tension controller in relation to the yarn tension significantly affects the quality of the resulting fabric, as proper tension control leads to strong, uniform, and aesthetically pleasing fabric, while poor tension control can cause defects and yarn breakage, leading to production downtime and increased costs. Maintaining the desired yarn tension during textile production is crucial, although it poses several problems, such as the continuous diameter change of the unwinder and rewinder sections leading to system change. Another problem faced by the industrial operation is maintaining proper tension on the yarn while changing the roll-to-roll operation velocity. In this paper, an optimized method for controlling yarn tension through the cascade control of tension and position, incorporating feedback controllers, feedforward, and disturbance observers, has been proposed to make the system more robust and suitable for industrial use. In addition, an optimum signal processor has been designed to obtain sensor data with reduced noise and minimal phase difference.

## 1. Introduction

The evolution of textile production from its origins in hand-weaving to contemporary automated systems involves a long and complex history that spans different regions and periods of the world [1]. A crucial element of modern textile production is the loom system [2], which enables the transformation of yarn into fabric by interlacing warp and weft threads. The loom system comprises a frame, a shedding mechanism, a reed, and a take-up mechanism. The frame secures the yarn, while the shedding mechanism creates a space between the warp and weft threads for the shuttle to pass through. The reed regulates the tension of the yarn, and the take-up mechanism collects the fabric as it is produced.

In the textile industry, weaving is a vital process [3] that demands meticulous attention to detail. One of the most important factors in this process is tension control, which refers to the capacity to adjust the tension of the yarn during weaving. The quality of the fabric produced by an industrial loom system is directly related to the level of tension control [4,5]. When the yarn is woven with optimal and consistent tension, the resulting fabric is durable, uniform, and aesthetically pleasing. On the other hand, poor tension control can cause fabric defects, such as irregularities in the weave, which compromise the quality of the final product. Tension control is also essential for preventing yarn breakage during weaving. When the tension of the yarn is not properly adjusted, it can snap or break, leading to production interruptions and increased costs. By maintaining the appropriate level of tension, the loom can operate efficiently and reduce the risk of yarn breakage.

To maintain the desired tension in the yarn, researchers face several problems [4], such as first of all if the reel continues with a fixed yarn velocity, the rotational velocity of the unwinder section will increase after a certain time because the yarn cake diameter of that section will reduce with the time. To avoid this problem, if the velocity of the rewinder increased initially, it may cause high tension on the yarn and result in a torn and bad product. Traditionally, to overcome this problem, mechanical controllers such as brakes or rotational weights are used. In addition, with the improvement of motor control techniques, scientists have tried to solve this problem through velocity control of the rewinder and unwinder motor. However, controlling tension with only the velocity of control generates a new problem due to the vibration [6] of the motor, which has been solved by adding a vibration controller with the velocity controller. In several studies, a tension control approach was adopted with the help of a load sensor, and in order to avoid the noise and disturbance generated by the sensor and system dynamics, the Kalman filter [7] and a disturbance observer were used. 

In this paper, we have studied a new method of controlling yarn tension through the cascade control [8,9] of tension as the outer loop and position as the inner loop. As the rolling velocity of the yarn is equally important as the yarn tension for mass production, this method will be helpful for industrial use. To reduce the noise generated by the load sensor, an optimized signal processor was designed with the help of the Kalman filter. In this study, each control loop [10] was structured with a feedback controller, a feedforward to eliminate phase delay and boost the system at the initial position, and lastly, a disturbance observer to make the system more robust so it can withstand dynamic change behavior. 

## 2. Roll-to-Roll Tension Modeling

In this section, we will conduct an analysis of the system modeling for roll-to-roll equipment. Additionally, we will propose a method for determining the system dynamics through experimentation and, subsequently, validate the optimal dynamics model.

### 2.1. System Dynamics Modeling

**Figure 1:**
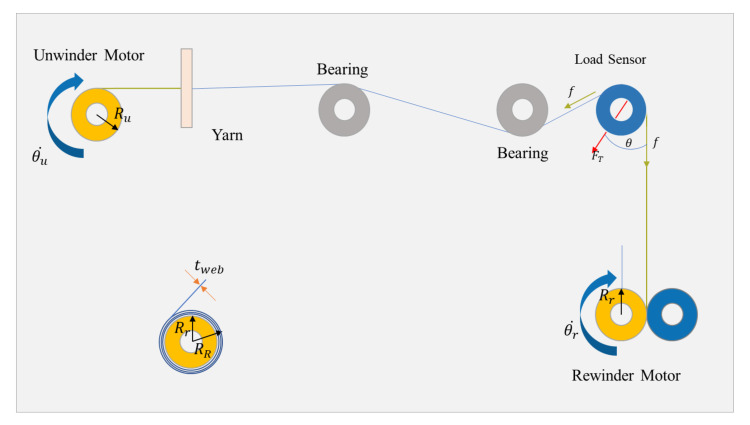
System dynamics outline.

In order to determine the system dynamics [11,12,13] of the roll-to-roll equipment, we have developed a schematic representation (Figure 1) that outlines the various components and stages involved in the roll-to-roll system. The yarn is initially unwound from the unwinder section at an angular velocity of $\dot{\theta_{u}}.$ Subsequently, it passes through several bearings and a single load sensor, before being fed into the rewinder section at an angular velocity of $\dot{\theta_{r}}$. As we are going to design the cascade control [14,15] of tension and position, we have to find a relation between them where the tension will be our input and we should obtain the position as an output from the system.

The load signal output $F_{T}$ varies depending on the web tension force f:

(1) $$F_{T}\left(t\right)=2f\left(t\right)cos\theta +F_{d}\left(t\right)$$

Here, $F_{d}$ is an external disturbance.

Again, we know that for the yarn elasticity, the tension force depends on the elasticity constant of the yarn and the movement from the steady position.

(2) $$f\left(t\right)=k\theta_{r}(t)$$

Thus, from Equation (1) we can find:

(3) $$F_{T}\left(t\right)=2k\theta_{r}\left(t\right).cos\theta =K\theta_{r}(t)$$

Now, the transfer function of the system from the input tension force ($F_{T}$) to the position output ($\theta_{r}$) is:

(4) $$P\left(s\right)=\frac{\theta_{r}\left(s\right)}{F_{T}\left(s\right)}=\frac{1}{K}$$

We posit that the physical distance between the actuator of the rewinder and unwinder and the load cell induces a signal delay in the system. To compensate for this delay, we suggest augmenting the nominal model with a dynamic model such as a first-order low-pass filter.

Thus, we can rewrite the nominal transfer function as:

(5) $$P_{n}\left(s\right)=\frac{1}{{K(\tau }_{n}s+1)}$$

### 2.2. System Identification for the Tension Model

**Figure 2:**
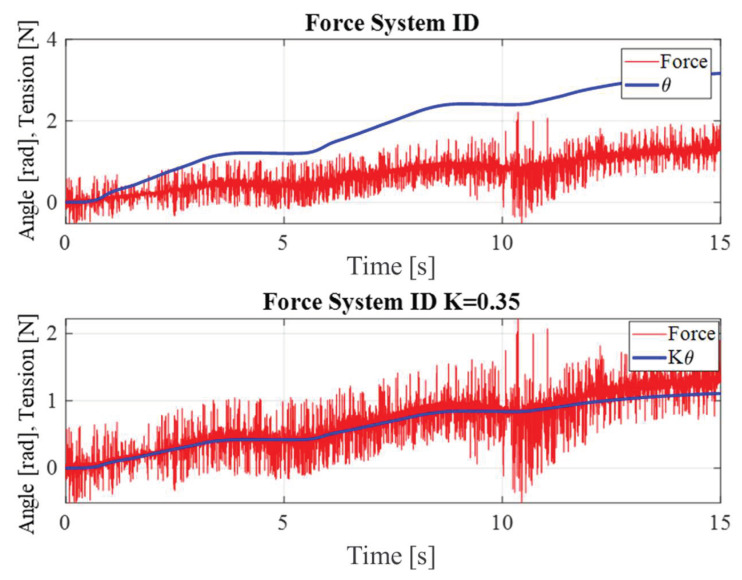
System identification of the tension model.

One of the essential steps in the model-based control design process is to obtain a suitable nominal model of the system. The quality of the nominal model affects the performance of the feedback, feedforward, and disturbance observer in the signal processing and control design, which will be elaborated in the subsequent sections. A step input with a varying position was applied to determine the dynamic model of the system, and the nominal model was derived from the load cell output data. The data sample was selected from the range of 0~1 N, which corresponds to the experimental scope, as shown in Figure 2.

### 2.3. System Identification for the Motor

**Figure 3:**
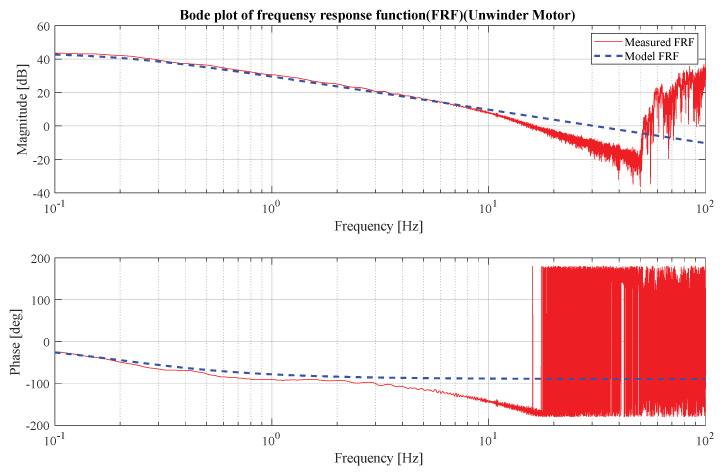
Unwinder motor Bode plot.

**Figure 4:**
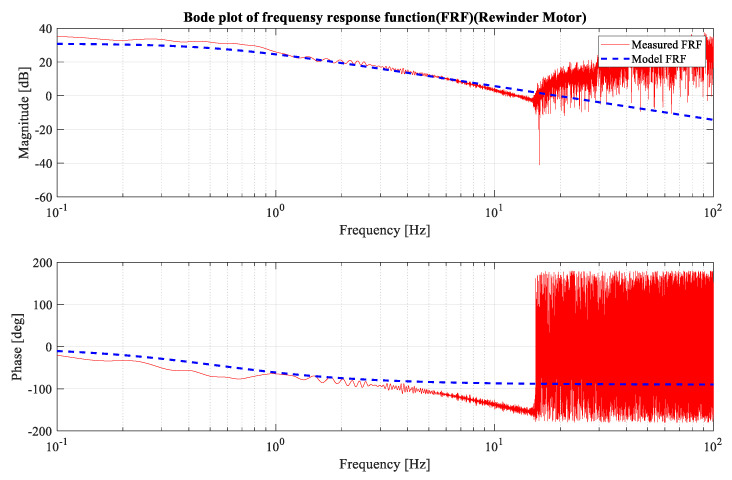
Rewinder motor Bode plot.

A model-based approach was employed to design the position controller, which necessitated the acquisition of an accurate nominal model of motor dynamics. A chirp signal with a frequency range of 0~50 Hz was applied for 50 seconds, and the output data were captured by the motor encoder. A fast Fourier transform was performed on the input–output data to facilitate the analysis, and a Bode plot of the motor system was produced. The Bode plot in Figure 3 and Figure 4 indicates that the system exhibited first-order dynamics.

## 3. Signal Processor

**Figure 5:**
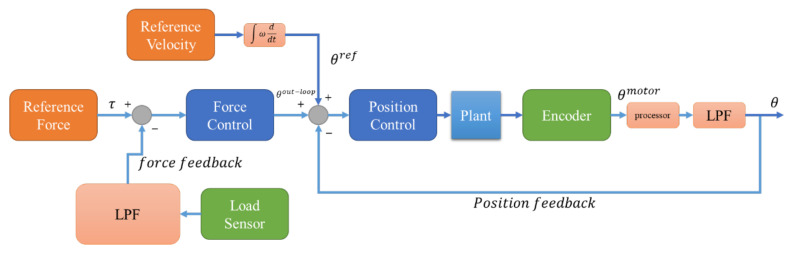
Overall control design of the experimental system.

As shown in Figure 2, the load sensor introduces a considerable amount of noise [16] that affects the precision of the control system. A noise filter can alleviate this problem, although it also introduces a trade-off between noise attenuation and phase lag. High noise attenuation may lead to a large phase lag and thus compromise the system’s stability. To address this challenge, this study developed an optimal Kalman filter that can achieve a desirable noise reduction within a tolerable range without sacrificing the phase lag. Figure 5 illustrate overall control architecture for the experimental system. The Kalman filter was designed by formulating the state space model of the tension nominal plant and estimating the state variables of the system through the Kalman filter using the measured values.

The transfer function of the system from the tension input τ to the load cell sensor output $F_{T}$ can be calculated as follows:

(6) $$f\left(t\right)=\frac{\tau \left(t\right)}{R_{r}}$$

From Equations (1)–(6), it can be expressed as:

(7) $$\frac{F(t)}{\tau (t)}=2cos\theta R_{r}+F_{d}$$

Acknowledging the system delay, the nominal plant can be written as:

(8) $$P_{tension}\left(s\right)=\frac{2cos\theta R_{r}}{\tau_{n}s+1}+F_{d}(s)$$

$$\tau_{n}=2*\pi *5$$

A state-space representation is required for the nominal plant in order to perform the Kalman filter computation. This involves expressing the system dynamics as a set of first-order differential equations that relate the state variables to the inputs and outputs of the plant.

(9) $$\dot{x}(t)=-\frac{1}{\tau_{n}}x\left(t\right)+\frac{2\cos\theta R_{r}}{\tau_{n}}u\left(t\right)+w\left(t\right)$$

(10) $$y\left(t\right)=x\left(t\right)+v\left(t\right)\left[Here,A=-\frac{1}{\tau_{n}},B=\frac{2cos\theta R_{r}}{\tau_{n}},C=1\right]$$

The Kalman filter algorithm [17] comprises two distinct stages, namely the prediction process and the correction process. To predict the future value of $\hat{X_{k}}$ as time progresses from $t_{k}$ to $t_{k+1}$, the algorithm utilizes the system model variables A and Q. A represents the system matrix, while Q denotes the covariance matrix of W. The system model variables, including A, Q, R, and H, significantly affect the performance of the Kalman filter. In this study, the system matrix A and the observation matrix H are assumed to be constant because they are related to the nominal model. The noise covariance matrices Q and R should be theoretically computed from the noise characteristics, although it is practically challenging to determine Q and R from multiple errors. Therefore, this study adopted a trial-and-error method to obtain Q and R. Equations (11) and (12) show the predicted variables of the Kalman filter algorithm.

(11) $$\hat{X_{k}^{*}}=A\hat{X_{k-1}}+Bu_{k}$$

(12) $$P_{k}^{*}=AP_{k-1}A^{T}+Q$$

The correction step of the Kalman filter involves computing the final estimate of the state vector ($\hat{x_{k}}$) based on the system model parameters H and R, the prior estimate ($\hat{x_{k}^{*}}$), and the current observation $Z_{k}$ multiplied by the Kalman gain $K_{k}$. The equation for the final estimate is given by:

(13) $$\hat{x_{k}}=\hat{X_{k}^{*}}+K_{k}\left(Z_{k}-H\hat{X_{k}^{*}}\right)$$

The calculation method for the Kalman gain $K_{k}$ is the following:

(14) $$K_{k}=P_{k}^{*}H^{T}{\left(HP_{k}^{*}H^{T}+R\right)}^{-1}$$

In this context, $P_{k}$ denotes the error covariance, which measures the discrepancy between the estimated and the true values. The error covariance can be computed as follows:

(15) $$P_{k}=\left(I-K_{k}H\right)P_{k}^{*}$$

**Figure 6:**
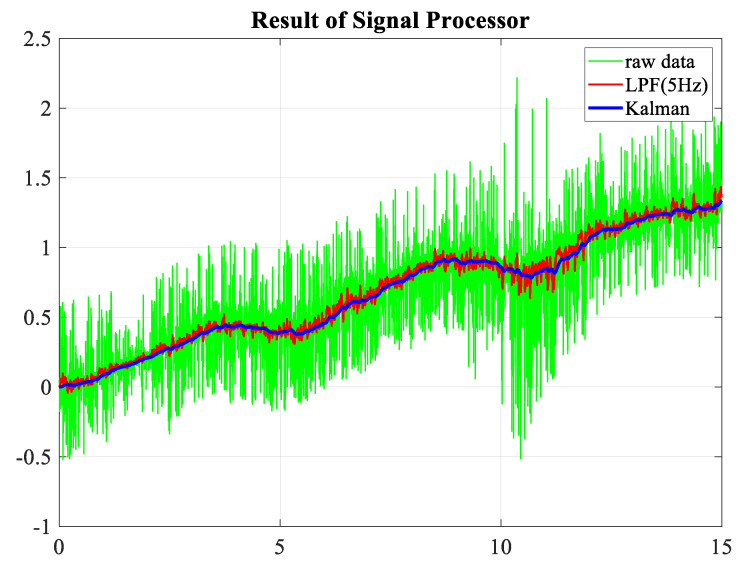
Kalman filter performance.

The algorithms presented in this paper were applied to the signal filtering problem, and the results are illustrated in Figure 6. The figure demonstrates the superior performance of the proposed methods over the conventional low-pass filter in terms of noise reduction and signal preservation.

## 4. Control Design

The aim of this research was to develop a precise and robust tension control system and to adjust the speed of the unwinder and rewinder motors for optimal looming performance. To achieve this, a cascade control [18,19] strategy was implemented, which integrated individual feedback loops for both tension and velocity as well as a disturbance observer (DOB) to enhance the robustness.

### Tension–Position Cascade Control Model

This study proposes a hierarchical cascade control architecture for a yarn tension control system. The architecture consists of two loops: an outer tension control loop and an inner position control loop. The outer loop receives the desired yarn tension as an input and generates a position signal to regulate the position of the inner loop. The inner loop controls the yarn tension by following the position signal from the outer loop, which is filtered with a low-pass filter to eliminate high-frequency noise. Additionally, the reference position for the inner loop is obtained by discretely integrating the reference velocity signal and adding it to the connection signal from the outer loop.

**Figure 7:**
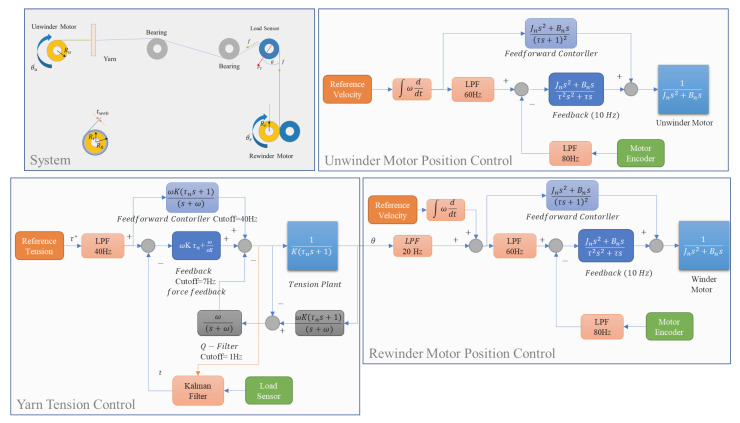
Tension–position control model.

The proposed cascade control design is applied to the rewinder motor, which requires precise regulation of the yarn tension. The unwinder motor uses an independent position control architecture that has the same reference input as the rewinder section. The details of this control scheme are presented in Figure 7, which includes the feedback, feedforward and disturbance observer design (with optimized gain [20,21]) for each loop. Moreover, a Kalman filter and several low-pass filters are integrated to suppress high-frequency noise and disturbance.

## 5. Experiment and Analysis

This section presents the experimental setup of the roll-to-roll process and the analysis of the performance of the proposed control architecture. The experimental setup consists of the components and parameters of the roll-to-roll system, while the control architecture includes the design and implementation of the control system discussed above.

### 5.1. Experimental Setup

**Figure 8:**
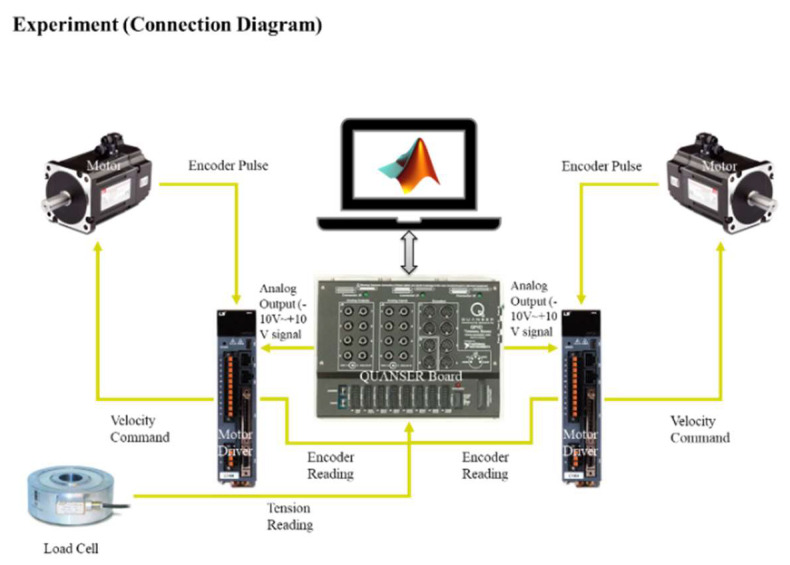
Hardware connection.

Figure 8 depicts the experimental configuration of the system. The rewinder and unwinder sections of the setup employed two servo motors (APMC-FAL01AM8K) with a power rating of 100 W, which were operated via the L7SA001 motor driver. The torque control mode of the motor driver was utilized to achieve compatibility with the control design framework presented in the previous section. The load sensor employed in the experiment was the M3200 model, which demonstrated the capacity to detect forces up to 50 N with adequate precision. The QUANSER board (a microcontroller provided by National Instruments; model: QPIDe) served as the primary control unit and was capable of providing an analog output of ±10 V [22] while offering a 19-bit resolution and high precision for the analog input, which meant a highly precious PWM [23] signal could be generated for the motor input. The QUANSER board was programmed using MATLAB and operated via the external mode option provided by MATLAB.

**Figure 9:**
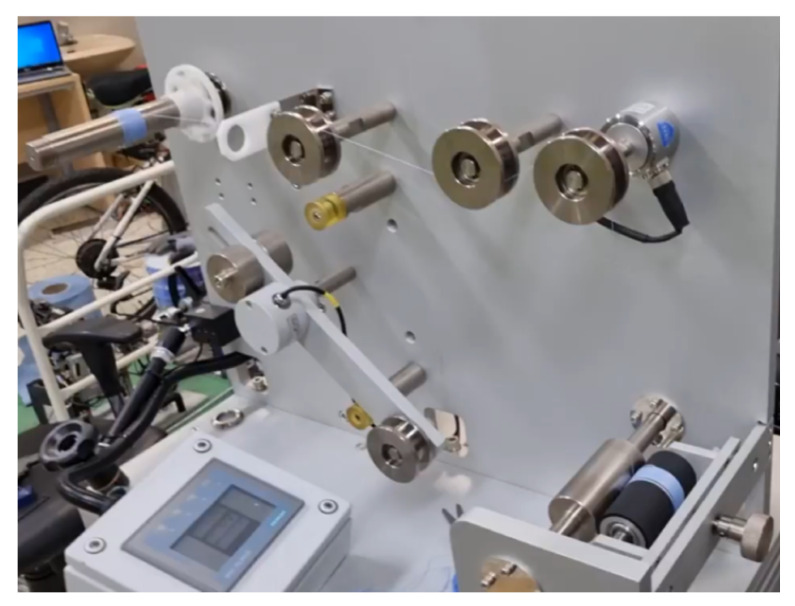
Hardware connection.

The real experimental setup is illustrated in Figure 9, which shows the positions of the rewinder and unwinder section and the load cell sensor.

### 5.2. Experimental Results and Analysis

**Figure 10:**
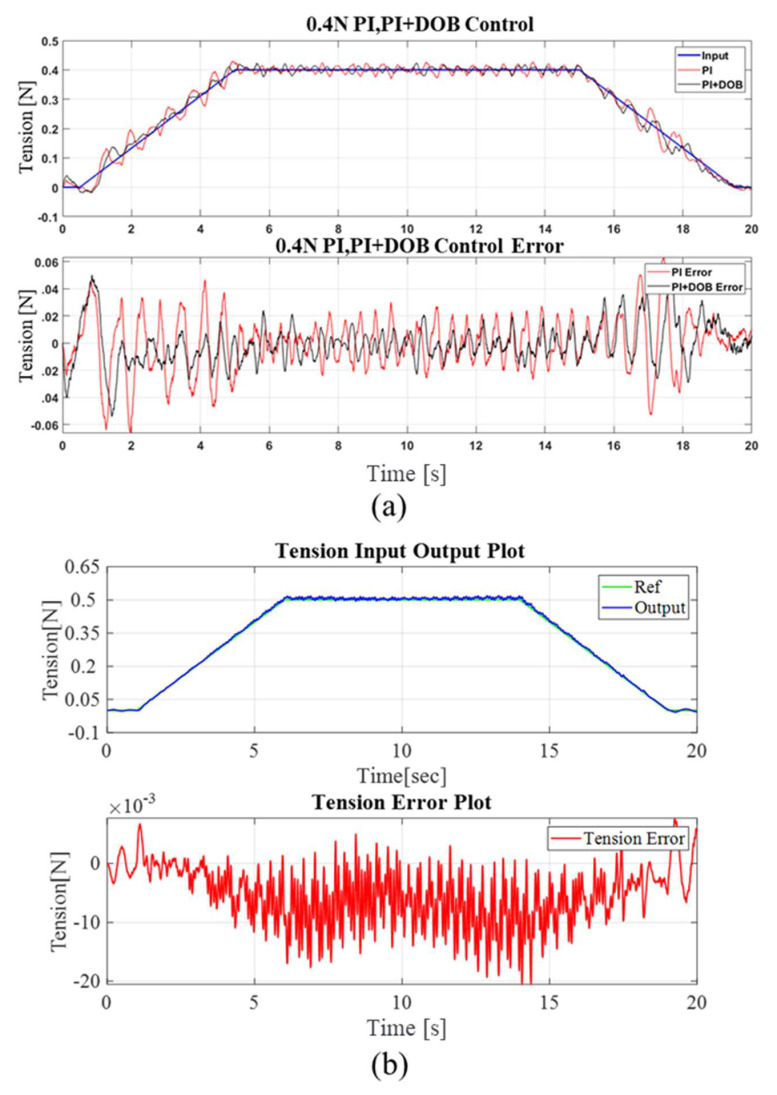
Comparison of control performance: (**a**) control performance without the Kalman filter, and (**b**) control performance with the Kalman filter.

To perceive the efficacy of the control design in terms of tension and position tracking, a trapezoidal tension profile input was applied to the system with a magnitude of 0.5 N. The velocity profile closely imitated the tension profile, with a maximum velocity of 19 rpm. It is noticeable that the velocity of the rewinder and unwinder motors differed, as the input profile of the rewinder motor was regulated via the tension control loop. The tension control performance of the outer loop with a proportional–integral feedback control scheme, without the use of a Kalman filter, is presented in Figure 10a. The results indicate that the tension performance was relatively poor, as the sensor noise had a direct impact on the performance. The inclusion of a disturbance observer led to a certain decrease in the tension error, as shown in Figure 10a. However, when the magnitude of the input signal increased or decreased, the tension error exhibited a significant increase, which is not particularly suitable for practical use. As soon as the Kalman filter was added as the load cell’s signal processor, the performance improved significantly, which is shown in Figure 10b.

**Figure 11:**
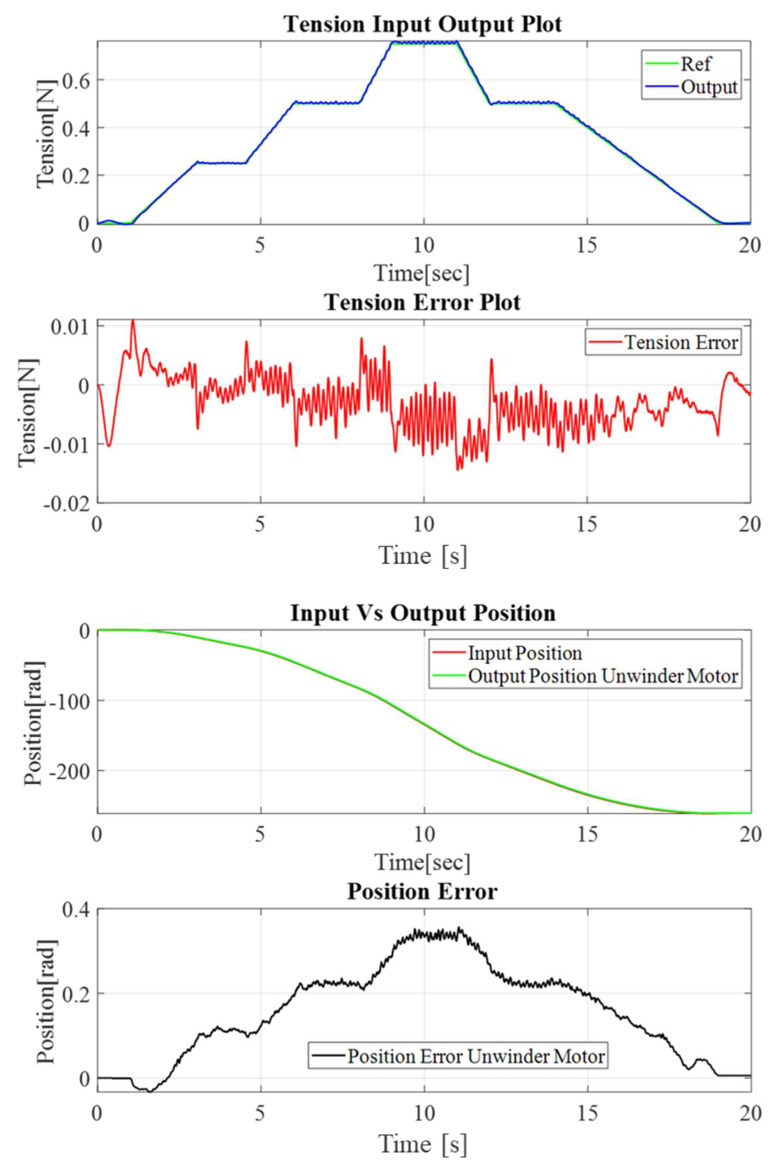
Tension–position control performance with a moderated profile.

As a further experiment, a gradually increasing trapezoidal tension profile, followed by a decreasing tension profile, were applied. The range of the tension profile varied between 0.1 N and 1 N. Figure 11 illustrates the control performance of the outer tension control loop as well as the inner loop’s position control performance.

**Figure 12:**
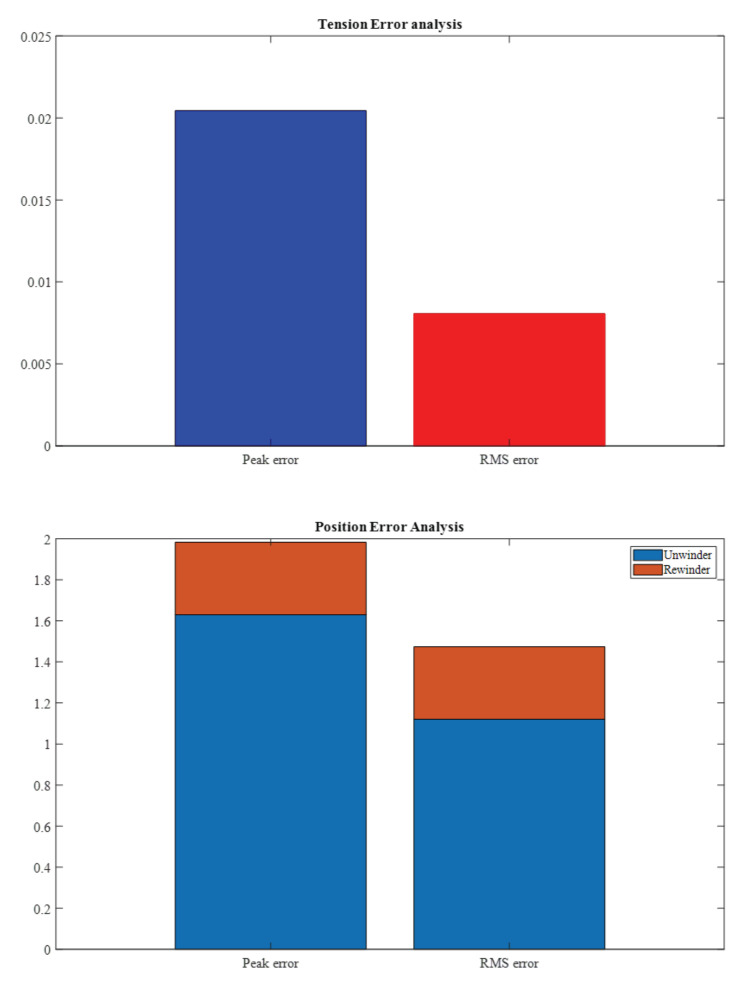
Error analysis of the control structure.

In Figure 12, a peak error of 0.02 N can be seen, while the RMS error is 0.008. However, for the inner loop position control, both the peak and RMS errors were high for the rewinder motor due to the processing time and phase delay of the cascade control loops.

## 6. Conclusions

The aim of this paper was to evaluate different performance measures for yarn tension control, rotational equipment position control, and sensor data processing. Tension control is a crucial factor in ensuring fabric quality, and thus, it received significant attention in this paper. Moreover, the velocity control of the rewinder and unwinder was also emphasized due to its relevance to the industrial production time. Considering the changing dynamics of the system, a robust tension–position cascade control method using a disturbance observer was proposed. The performance of each control loop was tested using real roll-to-roll experimental equipment, and the results were satisfactory for industrial use. Additionally, an adaptive signal processing filter was designed to eliminate sensor noise, which had a negligible phase delay. Future research could explore the use of reinforcement learning control algorithms to improve the performance of tension control at a more precise level.

## Data Availability

Not applicable.

## Appendix A. Symbols and Definitions

This appendix consists of a list (Table A1) of all the symbols used in this paper along with their definitions.

**Table A1:** List of symbols and their definitions.

Variable	Definition
$F_{T}$	Load sensor signal output
f	Tension force of the yarn
θ	Angle between the reference axis of the load sensor and the yarn
$F_{d}$	External disturbance
k	Elasticity constant of the yarn
$\theta_{r}$	Angular position of the rewinder motor
${\dot{\theta }}_{r}$	Angular velocity of the rewinder motor
$\theta_{u}$	Angular position of the unwinder motor
${\dot{\theta }}_{r}$	Angular velocity of the unwinder motor
$R_{r}$	Radius of the rewinder pully
$R_{u}$	Radius of the unwinder pully
$t_{web}$	Thickness of the yarn
$P_{n}$	Nominal plant of the system
$\tau_{n}$	Cutoff frequency of the nominal plant’s low-pass filter
τ	Input tension (reference)
s	Wheel radius
r	Roller radius
A	State matrix or system matrix
B	Input matrix
C	Output matrix
D	Feedthrough matrix
Q	Covariance of the process noise
R	Covariance of the observation noise
H	Observation matrix
$\hat{X_{k}}$	Estimated value at time k
$\hat{X_{k}^{*}}$	Predicted future value at time k
$Z_{k}$	Current observation at time k
$K_{k}$	Kalman gain at time k
$P_{k}^{*}$	Predicted error covariance at time k
$P_{k}$	Estimated error covariance at time k
I	Identity matrix
